# Dataset of STAT5A status in breast cancer

**DOI:** 10.1016/j.dib.2016.02.073

**Published:** 2016-03-04

**Authors:** Utpal K. Mukhopadhyay, Jamaica Cass, Leda Raptis, Andrew W. Craig, Véronique Bourdeau, Sonal Varma, Sandip Sen Gupta, Bruce E. Elliott, Gerardo Ferbeyre

**Affiliations:** a Université de Montréal, Département de Biochimie, Montréal, Québec, Canada H3C 3J7; b Division of Cancer Biology and Genetics, Queen׳s University Cancer Research Institute, Kingston, Ontario, Canada K7L 3N6; c Department of Biochemistry, Queens University, Kingston, Ontario, Canada

## Abstract

We analysed STAT5A gene expression in breast cancer using the Oncomine database. We exemplify four representative studies showing that STAT5A is generally downregulated in breast cancer.

## 1 **Specifications Table**

**Table:** 

Subject area	*Biology*
More specific subject area	*Cancer*
Type of data	*Figure*
How data was acquired	*Database Oncomine*[1]
Data format	*Filtered and analyzed*
Experimental factors	*Not applicable*
Experimental features	*We query Oncomine for STAT5A and breast cancer*
Data source location	*Montréal, Québec*
Data accessibility	*Data is within this article.*

## 2 **Value of the data**

**Fig. 1:**
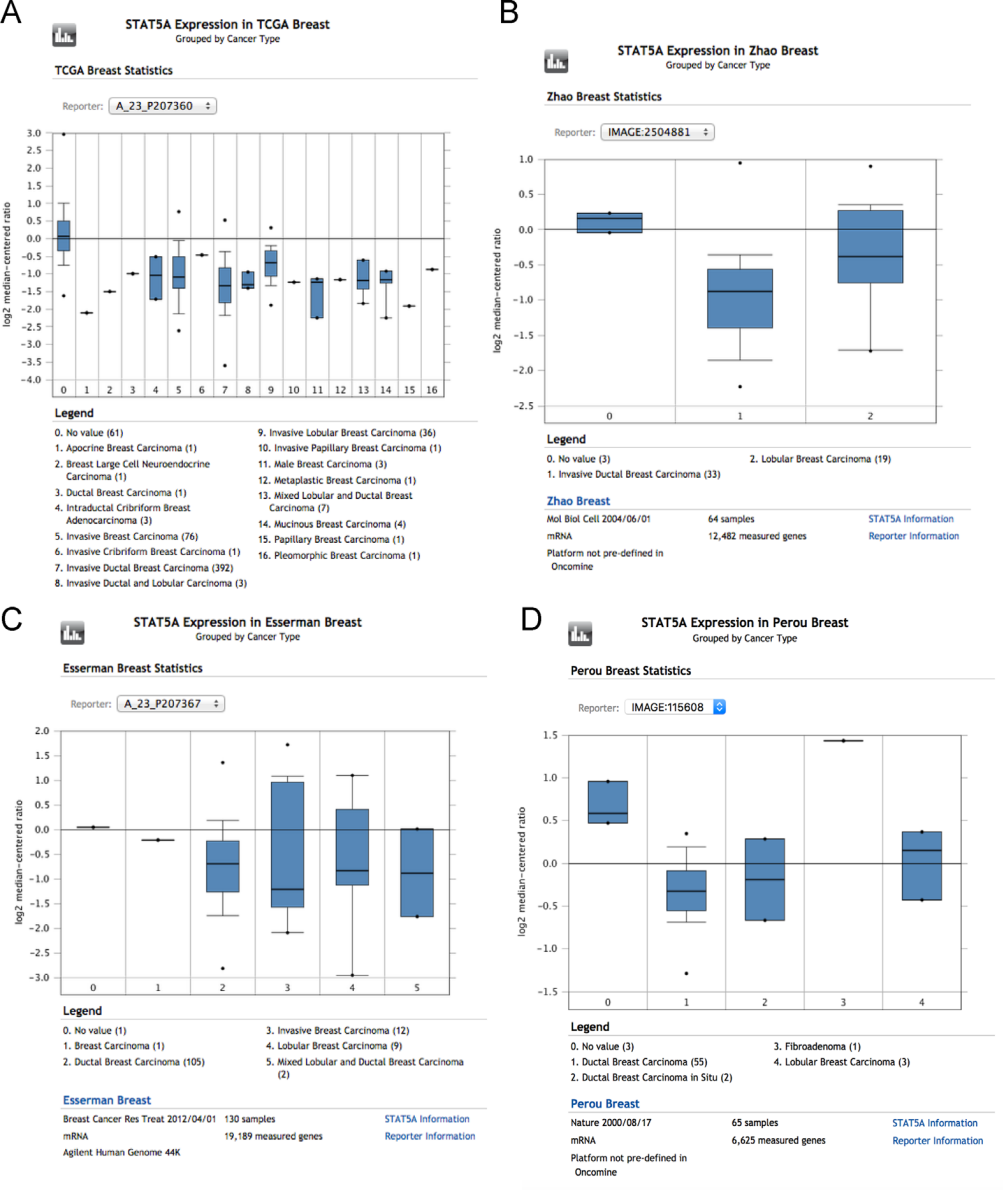
Downregulation of STAT5A expression in breast cancer (from Oncomine).

• We show that in different independent studies STAT5A is downregulated in breast cancer.
• The downregulation of STAT5A supports a tumour suppressor role for STAT5A in breast cancer and is consistent with our recent discovery that STAT5A is a p53-target gene [2].
• High levels of STAT5A indicate a good prognosis in breast cancer and it assessment can be used in the clinics as part of a multigene prognostic test (Fig. 1).

## 3 Data

We show that STAT5A is downregulated in breast cancer using several large-scale gene expression analyses available at Oncomine: https://www.oncomine.org/resource/main.html [1].

## 4 Experimental design, materials and methods

We used the publicly available Oncomine search to find the status of STAT5A in breast cancer.
